# A View on 20 Years of Antimicrobial Resistance in Japan by Two National Surveillance Systems: The National Epidemiological Surveillance of Infectious Diseases and Japan Nosocomial Infections Surveillance

**DOI:** 10.3390/antibiotics10101189

**Published:** 2021-09-30

**Authors:** Satowa Suzuki

**Affiliations:** Antimicrobial Resistance Research Center, National Institute of Infectious Diseases, Tokyo 187-0003, Japan; suzukiss@nih.go.jp

**Keywords:** antimicrobial resistance, surveillance, methicillin-resistant *Staphylococcus aureus*, multidrug-resistant *Pseudomonas aeruginosa*, vancomycin-resistant enterococci, multidrug-resistant *Acinetobacter* sp., carbapenem-resistant Enterobacterales (CRE)

## Abstract

The Ministry of Health, Labour and Welfare (MHLW) of Japan has conducted two national surveillance systems for approximately 20 years to monitor antimicrobial resistance (AMR) in bacteria: the National Epidemiological Surveillance of Infectious Diseases (NESID) and the Japan Nosocomial Infections Surveillance (JANIS). Data accumulated for 20 years by these two surveillance systems have helped depict the epidemiology of the representative AMR bacteria in Japan chronologically. The epidemiology of methicillin-resistant *Staphylococcus aureus* teaches us that once AMR bacteria have established their high endemicity, controlling such AMR bacteria requires time and is challenging. On the other hand, the epidemiology that multidrug-resistant *Acinetobacter* sp. exhibits when a strict containment policy for AMR bacteria was introduced in the early phase of its emergence and spread reveals that it is possible to control it. Detailed epidemiology provided by these two different national surveillance systems in Japan enabled us to set up the goal for controlling each AMR bacteria at the hospital level to the prefecture/national level. It is the public health authorities’ responsibility to maintain a good surveillance system for AMR bacteria and share the data and findings with healthcare professionals and academicians.

## 1. Introduction

Infectious disease surveillance is an eye to the public health. The fundamental epidemiology of infectious diseases can be understood using the surveillance data, which is critical to evaluate and plan the interventions needed to manage the target diseases. Antimicrobial resistance (AMR) is now recognized as a global public health issue. One of the five strategic objectives of the Global Action Plan of AMR is “strengthen the knowledge and evidence base through surveillance and research” [1]. 

Japan has two national surveillance systems implemented by the Ministry of Health, Labour and Welfare (MHLW) to monitor the trend of AMR bacteria, which have been in effect for the last 20 years. This review describes the epidemiology of representative AMR bacteria as causative pathogens of healthcare-associated infections based on these surveillance data. It will focus on methicillin-resistant *Staphylococcus aureus* (MRSA), multidrug-resistant *Pseudomonas aeruginosa* (MDRP), vancomycin-resistant enterococci (VRE), multidrug-resistant *Acinetobacter* sp. (MDRA), and carbapenem-resistant Enterobacterales (CRE) in Japan.

## 2. Surveillance Systems to Monitor AMR Bacteria in Japan

### 2.1. National Epidemiological Surveillance of Infectious Diseases (NESID)

#### 2.1.1. System Description

The current system of the National Epidemiological Surveillance of Infectious Diseases (NESID) has been operational since April 1999, when the “Law Concerning the Prevention of Infectious Diseases and Medical Care for Patients of Infections (Infectious Diseases Control Law)” was implemented. Since then, several amendments have been made to the Infectious Diseases Control Law, and 113 infectious diseases (as of August 2021) designated as “reportable infectious diseases” have been targeted under NESID. Those diseases are classified in the categories I to V based on the control measures and public health impact (Table 1). The control measures include restriction or blocking of traffic (Category I), recommend/order hospitalization (categories I and II), and restriction on attendance at work (categories I, II, and III). Infectious diseases in Category IV are mainly zoonotic diseases other than categories I to III. Category V includes infectious diseases that should be prevented by providing information on their prevalence to the public and medical personnel. Additionally, a different category termed “pandemic influenza and relevant infections” includes COVID-19. All cases of infectious diseases in the categories I–VI are required to be reported by the physicians promptly after the diagnosis. Two types of reporting are required for diseases belonging to category V: (a) all cases to be reported by the physicians within seven days after the diagnosis; and (b), to be reported by the sentinel clinics and hospitals on a weekly or monthly basis (sentinel surveillance diseases). The prefectural government selects those sentinel clinics/hospitals by considering the distribution of the prefecture’s population and medical facilities.

Cases of notifiable and sentinel surveillance diseases are first reported to the health center in a prescribed form. The health centers report the accepted cases to local public health authorities, usually at the prefecture level, through the NESID web-based system. The surveillance data are finally reported at the national level and released as the Infectious Diseases Weekly Report (IDWR) on the website of the National Institute of Infectious Diseases, Japan (https://www.niid.go.jp/niid/ja/idwr.html, accessed on 29 September 2021).

#### 2.1.2. AMR Pathogens 

As per surveillance definition, AMR is included for seven infectious diseases, all of which belong to category V (Table S1). Of them, three (MRSA, MDRP, and penicillin-resistant *Streptococcus pneumoniae* (PRSP)) are sentinel surveillance diseases reported by the sentinel hospitals on a monthly basis. At least one sentinel hospital exists in each medical region, and there are approximately 500 sentinel hospitals across Japan. The other four (VRSA, VRE, MDRA, and CRE) are notifiable diseases, for which all cases are to be reported and can be regarded as population-based surveillance. PRSP is an important AMR bacteria; however, because PRSP infection is a community-acquired infection rather than a healthcare-associated infection, a description of its epidemiology is out of the scope of this review. Additionally, no case of VRSA has been reported officially in Japan, and thus, there is no corresponding data of it.

### 2.2. Japan Nosocomial Infections Surveillance (JANIS)

Japan Nosocomial Infections Surveillance (JANIS) was launched in 2000 as a voluntary surveillance system focusing on infections in healthcare settings in Japan. JANIS is not based on the Infectious Diseases Control Law, but is funded by the MHLW and managed by the National Institute of Infectious Diseases. There are five divisions in the JANIS system with distinct surveillance objectives. Among them, the JANIS clinical laboratory division (JANIS-CL) is a surveillance system, which specifically focuses on AMR bacteria.

The details of the JANIS system have been described previously [2,3]. The unique feature of JANIS-CL is that it collects comprehensive specimen-based data from diagnostic microbiology laboratories of JANIS participating hospitals. The specimen-based data include patient demographics, sample type, isolated organism, and antimicrobial susceptibility test (AST) results. The type of specimen was initially limited to blood and cerebrospinal fluids, but it expanded to include all types of specimens in 2007. JANIS participating hospitals submit comprehensive specimen-based data directly to the JANIS system electronically on a monthly basis. The JANIS system releases the aggregated data at both the national and prefecture levels on the JANIS website (https://janis.mhlw.go.jp, accessed on 29 September 2021). 

Unlike the typical infectious disease surveillance, JANIS-CL does not include case definition for reporting. However, it defines each AMR bacteria (for example, MRSA, VRE, and CRE) based on species identification and AST results. Generally, these definitions are consistent with those defined by laboratory results per NESID case definitions. Conversely, the most important difference between NESID and JANIS-CL is that NESID cases include patients with symptomatic infections, whereas JANIS-CL includes both symptomatic and asymptomatic/colonized patients. Moreover, JANIS-CL division data cannot differentiate symptomatic and asymptomatic/colonized patients. For AMR bacteria, which are not targeted by NESID, the interpretation of AST results (susceptible or resistant) is mainly performed based on the Clinical and Laboratory Standards Institute (CLSI). 

The annual “Open Report” includes several types of aggregated data to assess the distribution of AMR bacteria. The prevalence of AMR bacteria in JANIS-CL is expressed as follows: the numerator denotes the number of patients with the specific AMR bacteria and the denominator denotes the number of patients whose clinical specimens for microbiological tests were submitted. The dissemination level of each AMR bacteria can be evaluated through assessing the proportion of hospitals reporting the particular AMR bacteria among all JANIS-CL participating hospitals. When this proportion is high, the corresponding AMR bacteria is considered endemic and disseminated widely. Conversely, when it is low, the dissemination of the corresponding AMR bacteria is considered limited. Finally, the antimicrobial susceptibility pattern based on bacterial species, usually called antibiograms, has also been depicted for major bacterial species. 

The number of JANIS participating hospitals has increased since 2007, and as of January 2021, more than 2000 hospitals are included in JANIS-CL. It accounts for almost a quarter of all hospitals (approximately 8300 hospitals exist in Japan). Additionally, focusing on larger hospitals with more than 500 beds, which usually include the tertiary care, general, or teaching hospitals, more than 80% of those hospitals are participating in this surveillance system. Therefore, JANIS-CL division data have good representation, especially of the tertiary care hospitals in Japan.

## 3. Epidemiology of AMR Bacteria in Japan

### 3.1. MRSA

**Figure 1:**
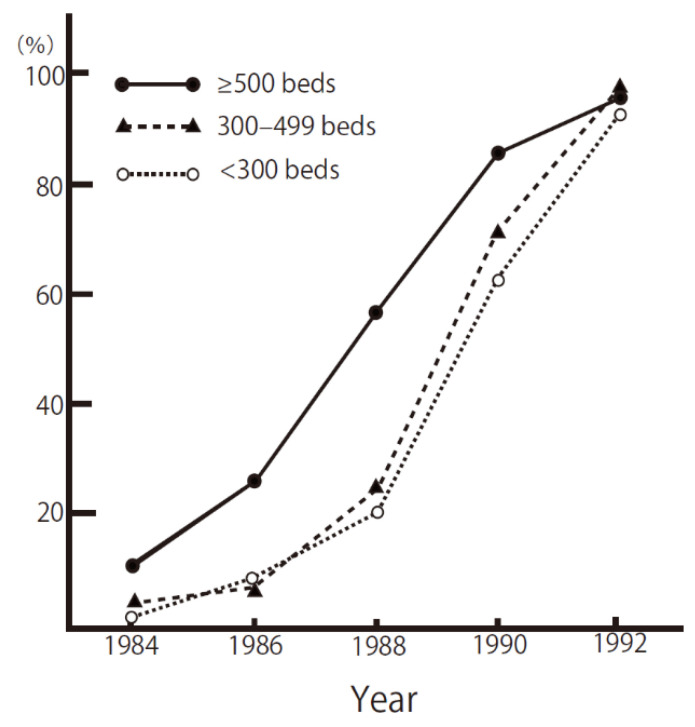
Proportion of hospitals with MRSA isolation, 1984–1999, based on a questionnaire survey of hospitals in Japan. Translated into English from reference [4] with permission.

MRSA is a representative AMR bacteria in Japan, and its emergence and rapid spread have made Japanese people realize the importance of infection control in healthcare settings, not only by the healthcare workers, but also by the general public. Figure 1 indicates the rapid rate at which MRSA disseminated in Japan during its epidemic in the 1980s, spreading from large to medium to small hospitals. It has been reported that, in the early 1990s, 70% of *S. aureus* isolated from the clinical specimens were MRSA [4]. MRSA is still isolated in almost all the Japanese hospitals and is one of the most ubiquitous AMR bacteria in Japan. 

**Figure 2:**
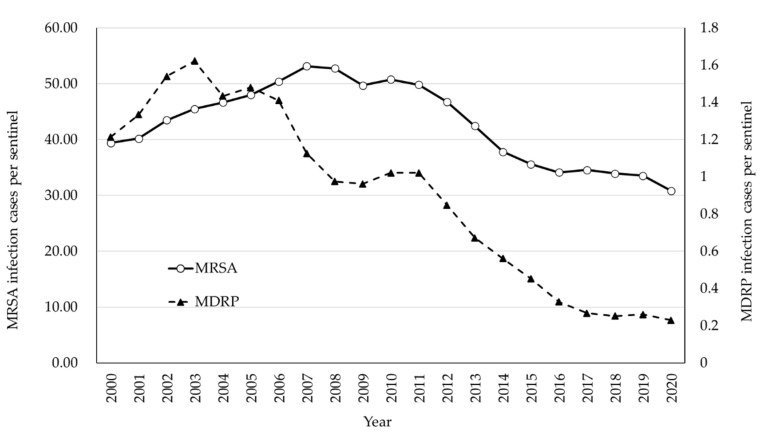
Number of reported cases of MRSA and MDRP infection per sentinel, NESID 2000–2020.

MRSA infection cases reported to NESID per sentinel are shown in Figure 2. The incidence of MRSA infections has exhibited an increasing trend until it started decreasing in the 2010s. Although infection control has been strengthened in hospitals since the MRSA epidemic began in the 1980s, it was difficult to control MRSA transmission, which had already established its endemicity in Japanese medical settings. Additionally, there were not enough trained infection control professionals at that time, and the need and importance of infection control measures were not very well understood by the hospital administrators. The decreasing trend may be partly due to the introduction of reimbursement of medical fees against the infection control practices at hospitals in 2010. Hospitals fulfilling the requirements of infection control practices were eligible to receive a reimbursement of 1000 Yen (9 to 10 USD) per patient per admission. This was a significant and enough economic incentive to hire a full-time certificate infection control professional [5]. This reimbursement was increased in 2012 to 4000 Yen per patient per admission. It is also important to note that a large number of infection control professionals were deployed at that time.

In spite of these policies and developed human resources, the decrease in MRSA incidence slowed down in the late 2010s. A possible reason could be inferred from JANIS-CL data. Unlike NESID, which includes data only from sentinel hospitals (usually tertiary care hospitals with more than 300 beds), JANIS-CL receives data from small hospitals with less than 200 beds. In Table 2, the comparison of AMR bacteria prevalence between large and small hospitals is shown. It can be clearly seen that MRSA prevalence was much higher in small hospitals than that in large hospitals, and it was in contrast to that of other AMR bacteria. 

The MRSA epidemic started from large hospitals and rapidly spread to small hospitals by transferring patients (Figure 1), and it was regarded as an endemic for more than 30 years. It is likely that large pools of MRSA-colonized patients exist in non-acute care, and small hospitals, and may continuously spread MRSA to the tertiary care hospitals. 

It is noteworthy that the reimbursement of 4000 Yen per patient per admission for implementing infection control practices is mainly targeted at the large hospitals. Conversely, of the 8300 hospitals in Japan, 5769 (69.5%) are small hospitals with less than 200 beds. Usually, the small hospitals are privately owned and provide both acute- and long-term care. Smaller reimbursement (1000 Yen per patient per admission) for implementing infection control practices in the small hospitals also exists with minimal requirements. Infection control in mixed or long-term care facilities with limited resource needs different strategies than in acute care hospitals, making it an urgent and important issue requiring intervention in Japan.

### 3.2. MDRP

In Japan, MDRP is defined as *P. aeruginosa* resistant to carbapenems, fluoroquinolones, and amikacin (Table S1). The emergence of MDRP in Japanese medical settings with multifocal outbreaks was firstly reported in the 1990s, which made MDRP infection one of the target AMR bacteria to be considered for national surveillance [6]. NESID data indicate that the MDRP epidemic in Japan lasted from the early to mid-2000s, although the number of reported cases per sentinel was far less than that of MRSA (the peak of reported cases of MDRP was 1.62/sentinel in 2003, while that of MRSA was 53.15/sentinel in 2007) (Figure 2). During this epidemic period, outbreaks in general and teaching hospitals were reported [7,8,9,10,11]. MDRP isolates responsible for these outbreaks usually produced IMP-type metallo-β-lactamase, which is an endemic carbapenemase in Japan. Clinically, MDRP was most frequently isolated from the urinary tract samples (43.8% of MDRP isolates) and 34.2% were from the respiratory tract. Conversely, for all *P. aeruginosa* isolates, the respiratory tract is the most frequent source, while 17.0% and 55.3% of isolates were isolated from urinary and respiratory tracts, respectively [12]. 

Measures have been taken to contain the MDRP epidemic based on the finding of the outbreak investigations. Strengthening the contact precaution, isolation of patients with MDRP, improvement of medical device management, and environmental cleaning were promoted [8,10,11,13]. One of the unique interventions was to stop urine collection practice [9]. To measure and record the urine volume per day using a shared automatic urine collection machine located at the ward bathroom was a common practice at the Japanese hospitals. Both medical staff and patients, who could walk and go to the ward bathroom, were requested to use this machine, and its contamination was suspected as a source of MDRP outbreak. 

**Figure 3:**
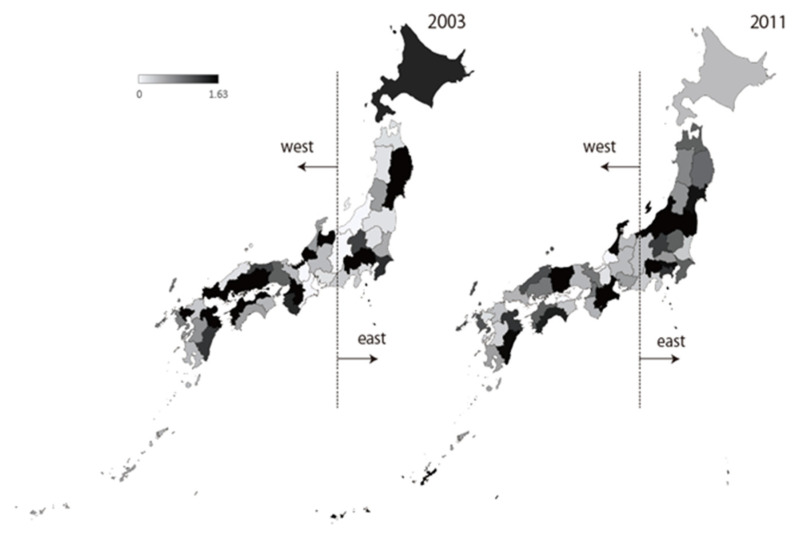
Reported case of MDRP infection per sentinel, NESID 2003 and 2011.

The number of reported cases per sentinel exhibited a decreasing trend after the epidemic from the early to mid-2000s. However, a slight increase was recorded in 2010 and 2011. Interestingly, during the first epidemic, the prefectures in the west part of Japan reported more MDRP cases, but in 2011, the prefectures in eastern Japan reported more MDRP cases (Figure 3). It is noteworthy that the emergence and spread of a novel MDRP strain were reported at that time [14]. Moreover, the proportion of MDRP was high in central to eastern Japan compared to western Japan in 2011 and 2012 [15], which is in agreement with NESID data presented in Figure 3. Furthermore, an increasing number of MDRP isolates were reported from the respiratory tract samples [15], which is in contrast to the urinary tract samples during the early to mid-2000s. 

**Figure 4:**
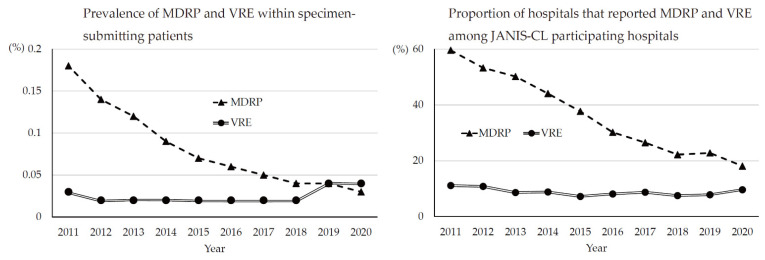
Trends of MDRP and VRE in JANIS-CL, 2011 to 2020.

Fortunately, regardless of the emergence of a novel MDRP strain, reports of MDRP cases dramatically decreased in the 2010s, from 1.02 cases/sentinel in 2011 to 0.23 cases/sentinel in 2020. This dramatic decrease may be attributed to the strengthening of the infection control policy with reimbursement, as mentioned for MRSA. It appears that the decrease in the numbers of MDRP cases also reduced in late 2010s, especially after 2017. Because of the low incidence, it became difficult to assess the trend based on the sentinel surveillance of NESID. However, JANIS-CL data may complement this issue (Figure 4). The decrease in MDRP prevalence as per JANIS is clearer as it was 0.05% in 2017 and 0.03% in 2020. Furthermore, in 2017, 26.5% of JANIS-CL participating hospitals reported MDRP, which decreased to 18.1% in 2020.

### 3.3. VRE

**Figure 5:**
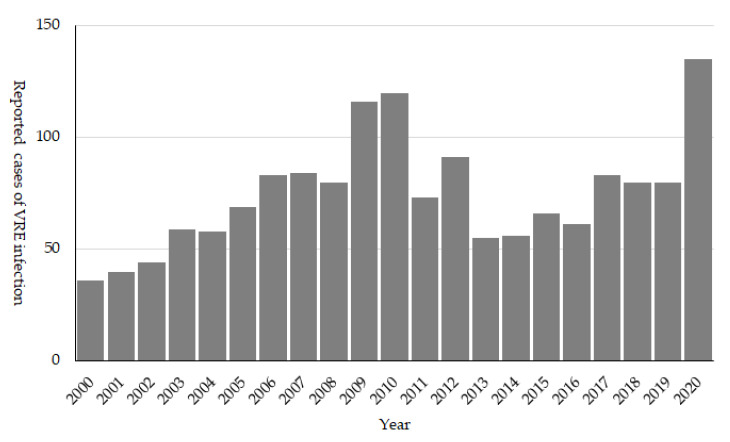
Number of reported cases of VRE infecion, NESID 2011 to 2020.

VRE was first reported in 1996 from a clinical specimen in Japan [16], and due to the concern for the subsequent increase in the cases of VRE infection, it was designated as a notifiable disease in 1999. The number of reported cases first peaked in 2009 to 2010, when around 100 cases were reported annually (Figure 5). During the 2000s, several hospital outbreaks were reported, and including hospitals in the same medical region extended their support to control VRE thorough promoting active screening and strengthening the infection control policy [17,18,19]. The strong policy against VRE from its emergence seems to have resulted in successful control of VRE, and it is still considered as a rare AMR bacteria in Japan.

**Figure 6:**
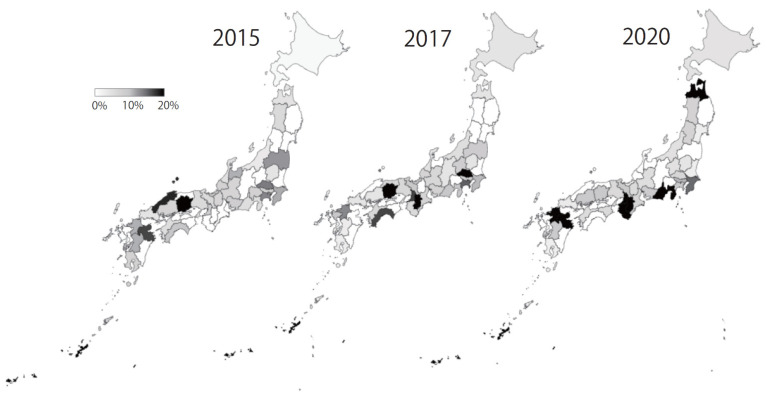
Proportion of hospitals with VRE isolates among JANIS participating hospitals based on prefecture level, 2015, 2017 and 2020.

In JANIS-CL, the prevalence of VRE was almost stable (0.02%) in the 2010s; however, it increased to 0.04% since 2019 (Figure 4). Of note, VRE prevalence was slightly higher than that of MDRP (0.03%) in 2020, but the proportion of hospitals reporting VRE (9.6%) is lower than that of MDRP (18.1%). This discrepancy may be due to the fact that VRE is intensively isolated in epidemic prefectures, while MDRP is more sporadic than VRE. Among JANIS participating hospitals, the proportion of hospitals, which reported VRE isolates based on the prefecture, varied from 0% (which means no VRE isolation at hospitals in the prefecture) to more than 40% in 2020. Interestingly, those highly endemic prefectures were not fixed to certain areas but changed over the years (Figure 6). This indicates that, once a VRE outbreak occurs, it can rapidly spread within the same medical region. However, even if VRE can disseminate to multiple hospitals within the medical region, it is controllable and possible to prevent it from establishing endemicity. 

Since 2019, several VRE outbreaks with more than 100 cases per hospital have been reported, which is unusual in Japan. These VRE outbreaks started from a single hospital but spread rapidly within the entire medical region. Both NESID and JANIS-CL data reflected these outbreaks, and their rapid dissemination as the number of reported VRE cases recorded were the highest in 2020, and an increase in VRE prevalence was recorded in JANIS-CL (Figure 4 and Figure 5). The reason for the second epidemic in Japan is still unclear; however, the recent increase in VRE prevalence was reported worldwide and some association with emerging high-risk clone (ST1420-like or *pstS*-null *Enterococcus faecium*) first reported in Australia is suggested [20,21,22,23]. It is important to carefully monitor the VRE surveillance data and investigate the hospital and local outbreaks to support the implementation of effective control measures.

### 3.4. MDRA

**Figure 7:**
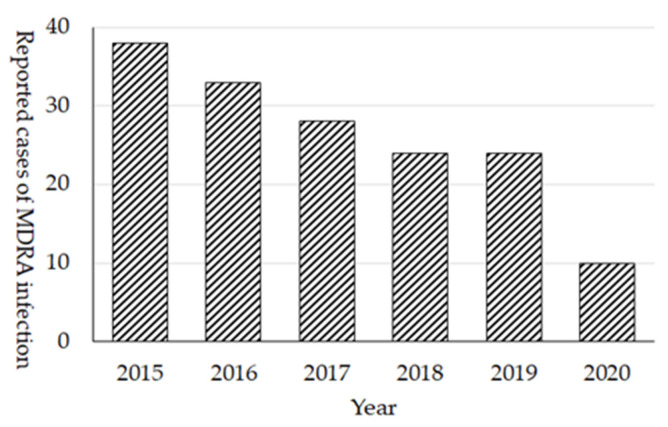
Number of reported cases of MDRA infecion, NESID 2015 to 2020.

The prevalence of MDRA in Japan still remains quite low. Cases of MDRA infection were initially (February 2011) designated as sentinel reporting diseases in NESID; however, because of the low prevalence, it was changed to notifiable diseases in September 2014. In 2015, 38 cases were reported and the number of cases continuously decreased as there were only 10 cases reported in 2020 (Figure 7). Among 172 cases of all reported cases of MDRA, at least 25 (15%) cases were reported as the import cases from overseas, mainly from the Asian countries. In regard to the extremely high prevalence of MDRA in other countries, preventing the local transmission of MDRA from the imported cases is essential in Japan. In fact, the dramatic decrease in reported MDRA cases in 2020 (10 cases) and 2 cases in 2021 (as of August 2021) after the COVID-19 pandemic was observed, it may be attributed to the cessation of international travel. 

In the 2020 JANIS-CL annual report, the prevalence of MDRA was 0.003% (92 patients out of 1,757,567 specimens-submitting patients), reflecting the low prevalence, and only 21 (1.0%) hospitals out of 2167 JANIS participating hospitals reported MDRA.

Although the dissemination of MDRA in Japan is limited, it did play an important role in implementing the infection control policy. In 2010, an outbreak of MDRA with 53 cases including nine fatalities occurred [24]. The hospital did not report its outbreak to the local health center, because there was no clear rule for reporting the outbreaks in healthcare settings at that time. It was reported by mass media, and consequently, police investigation began on suspicion of manslaughter. The involvement of the police department in nosocomial outbreaks due to AMR bacterial was quite unusual and raised concerns. This outbreak triggered the establishment of rules for reporting hospital outbreaks to the health center, as well as promoting the involvement of the public health sector in hospital outbreak management, and strengthening the laboratory capacity to enable appropriate testing for pathogens causing healthcare-associated infection, which are usually AMR bacteria, at local health laboratories. This policy was announced in 2011 by the MHLW [25].

### 3.5. CRE

The rapid dissemination of NDM-type carbapenemase-producing Enterobacterales (CPE) reported in 2010 made CRE widely recognizable as an AMR pathogen of concern. This was also reported by the Japanese mass media together with the first report of NDM-type CPE isolation in Japan [26]. CRE infection was designated as a notifiable disease in 2014, and pathogen surveillance began in 2017 to distinguish CPE and non-CPE among CRE. CRE was the first AMR bacteria introduced in the national pathogen surveillance in NESID, which means that the local health laboratories conducted tests for the presence of carbapenemase genes in CRE isolates from reported cases. National pathogen surveillance for CRE was possible to launch, because laboratory capacity for testing AMR bacteria in local health laboratories was established by then. It was partly due to the policy announcement made in 2011, as described previously.

**Figure 8:**
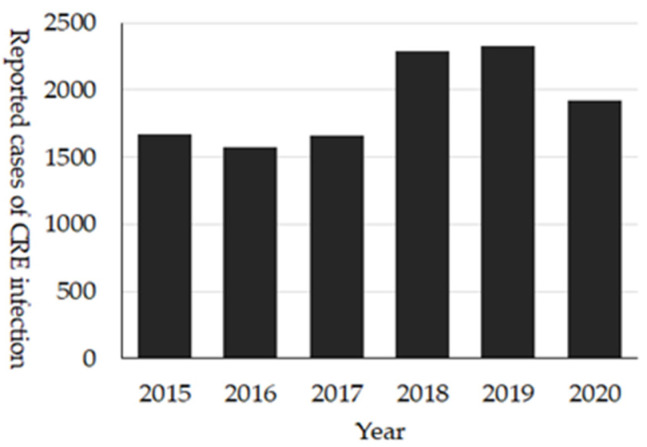
Number of reported cases of CRE infection, NESID 2015 to 2020.

These comprehensive national surveillance systems reveal the unique epidemiology of CRE in Japan. Firstly, around 2000 cases of CRE are reported annually (Figure 8), and of them, about 15–30% were cases with CPE [27]. The most prevalent bacterial species among CRE is *Klebsiella aerogenes,* while that among CPE is *Enterobacter cloacae* complex. Additionally, IMP-type is the dominant carbapenemase. The dominance of IMP-type carbapenemase is different from Europe, the United States, or other Asian countries where OXA-48-like, KPC- or NDM-type CPE are more dominant types of carbapenemase. In Japan, those types of CPEs are thought to be an imported-type of AMR. However, for NDM-type CPE, cases without international travel have been reported, which is suggestive of local transmission. The dominance of *K. aerogenes* may be due to the unique definition of CRE in Japan (Table S1).

In the 2020 JANIS-CL annual report, the prevalence of CRE was 0.31%, and 51.2% of hospitals among JANIS participating hospitals reported CRE. One of the limitations of JANIS-CL data of CRE is that it is impossible to distinguish CPE from non-CPE. 

## 4. Benchmarking AMR Bacteria in Japan 

Comparing the AMR rate internationally has several issues to be addressed. The WHO Global Antimicrobial Resistance Surveillance System (GLASS) [28], which defined the reporting methods, is one of the solutions. However, the target and objectives of AMR surveillance may differ for each country. The socioeconomic status, healthcare systems, laboratory capacity, and quality of infection control practices may affect the epidemiology of AMR bacteria. Consequently, they also affect the risk assessment of those AMR pathogens for prioritizing and selecting the surveillance target as well as choosing the most feasible surveillance method. For instance, VRE is not included as a target pathogen in GLASS; however, it is listed as one of the most important AMR bacteria in Japan, as it is a notifiable disease included in Category V based on the Infectious Diseases Control Law.

Both NESID and JANIS-CL are AMR surveillance systems, which were optimized for Japan. Therefore, they were sustained for more than 20 years with verified data and high representativeness. However, the number of reported cases of AMR bacterial infections in NESID and prevalence of AMR bacteria calculated in JANIS-CL is hardly comparable with those of other countries. It is because the definitions of AMR bacteria are different (different antimicrobials as resistance indicator and different breakpoint of them for resistance) and different denominators, such as the number of specimens-submitting patients, as used in JANIS-CL.

**Figure 9:**
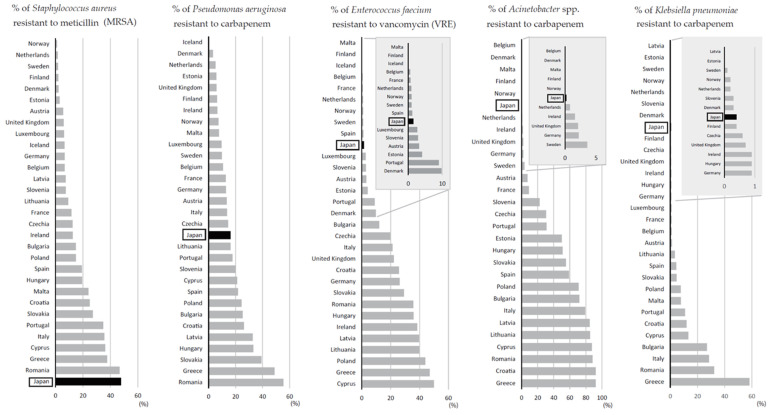
Comparison of resistance rates between Japan (2019 JANIS-CL annual report) and European countries for five bacterial species in 2019 [29]. The black bars indicate Japan (enclosed in rectangles), while the gray bars indicate European countries. Countries with very low resistance rates compare to those with the highest countries are magnified by a small insert with a grey background. The graphs are arranged in order of the epidemic/spread of each AMR bacteria in Japan: MRSA, 1980s to 2000s; MDRP (carbapenem-resistant *P. aeruginosa*), 1990s to mid-2000s; VRE (vancomycin-resistant *E. faecium*), 2000s; MDRA (carbapenem-resistant *Acinetobacter* spp.), late 2000s to early 2010s; CRE (carbapenem-resistant *K. pneumoniae*), 2010s.

Conversely, data for antibiograms in the JANIS-CL division, which indicate antimicrobial-resistant rate based on the bacteria species for several antimicrobials independently, are more comparable internationally. As described previously, it follows CLSI for SIR (susceptible, intermediate, and resistant) interpretation. Figure 9 shows the comparison of resistance rate between Japan and European countries for five bacteria species to benchmark the prevalence of five major AMR bacteria (MRSA, MDRP, VRE, MDRA, and CRE) in Japan [29]. For this purpose, carbapenem resistance rate for *P. aeruginosa* and *Acinetobacter* spp. was adapted instead of MDRP and MDRA, respectively, and *E. faecium* was considered as a representative bacterial species of VRE. For CRE, *K. pneumoniae* was used as a representative bacterial species, because it is the most prevalent bacterial species in European countries, although it is not the most dominant bacterial species in Japan. Those comparing charts were organized by the period of their epidemic or emergence/spread.

It seems that Japan has succeeded in controlling AMR bacteria causing healthcare-associated infections which emerged and spread after the 2000s, such as VRE and MDRA. This could be accomplished because of the improvement of infection control practices, and training and deployment of infection control professionals in the 2000s [5]. However, even with these well-established infection control practices in Japanese medical settings, it is difficult to reduce MRSA, which has been widely disseminated for more than 30 years. This emphasizes the importance of containing AMR bacteria in its early phase of emergence. Therefore, it is especially important in Japan to continue the efforts to maintain low prevalence of VRE, MDRA, and CRE.

## 5. AMR Surveillance Data under the COVID-19 Epidemic in Japan

The COVID-19 epidemic, which started in early 2020, has induced drastic behavioral changes in people and caused a significant decrease in other infectious diseases like influenza. The behavioral changes may also include healthcare-seeking behavior. The number of hospitalized patients per day decreased by almost 7–8% since the COVID-19 epidemic. It can also be seen in JANIS data, as the number of patients submitting specimens decreased by 7.2% in 2020 compared to 2019. Furthermore, reports of COVID-19 outbreaks in healthcare settings may have strengthened infection prevention practices, especially standard and droplet infection precautions. Although more careful study is required, these may explain the slight decrease of MRSA, MDRP, and CRE cases in both NESID and JANIS in 2020. In terms of MDRA, the decrease in the reported cases in NESID may be largely attributed to the decrease in imported MDRA cases from overseas.

Conversely, as described in VRE section, Japan is now facing the nationwide epidemic of VRE. This may indicate that, to contain VRE dissemination, more pathogen-focused infection control measures, including strengthening contact precaution, active surveillance to detect VRE colonized patients combined with adequate isolation and cohorting, are required. Additionally, even if regional VRE outbreaks were detected, it was difficult for health centers and hospitals to focus on VRE outbreak management during the COVID-19 epidemic. The careful assessment of AMR surveillance data during and after the COVID-19 epidemic is important for controlling AMR in the future.

## 6. Conclusions

It has been more than three decades since the Japanese medical setting has been facing an MRSA epidemic as a cause of healthcare-associated infections and two decades since the surveillance systems to monitor AMR bacteria were launched. The accumulation of nationwide, representative surveillance data of AMR for the past 20 years in Japan has contributed to our knowledge of AMR epidemiology, which is applicable for evidence-based intervention. AMR bacteria are sometimes described collectively. However, specific AMR bacteria, such as MRSA, MDRP, and VRE, exhibit different epidemiology, and therefore, the focus point for intervention can be different. Therefore, healthcare professionals in this field need to understand the importance of surveillance and cooperate in surveillance data collection. In addition, it is a responsibility of the public health authorities to maintain a good surveillance system for AMR bacteria and share the data and findings with the healthcare professionals and academicians to improve evidence-based infection control measures.

## Figures and Tables

**Table 1:** Disease categories of the National Epidemiological Surveillance of Infectious Diseases (NESID).

Category *	Number of Diseases	Infectious Diseases
I	7	Ebola hemorrhagic fever, Plague, etc.
II	7	Acute poliomyelitis, Diphtheria, Tuberculosis, etc.
III	5	Cholera, Typhoid fever, Enterohemorrhagic *Escherichia coli* infection, etc.
IV	44	Anthrax, Botulism, Dengue fever, Japanese encephalitis, Yellow fever, etc.
V (notifiable diseases)	24	Acute immunodeficiency syndrome (AIDS), Rubella, Tetanus, etc. (AMR pathogens) Vancomycin-resistant enterococci (VRE) infection, Vancomycin-resistant *Staphylococcus aureus* (VRSA) infection, Multidrug-resistant *Acinetobacter* spp. (MDRA) infection, Carbapenem-resistant Enterobacterales (CRE) infection
V (sentinel surveillance diseases)	25	Weekly: Influenza, Mumps, RS virus infection, etc. Monthly: Gonorrhea, Genital chlamydial infection, etc. (AMR pathogens) Methicillin-resistant *Staphylococcus aureus* (MRSA) infection, Multidrug-resistant *Pseudomonas aeruginosa* (MDRP) infection, Penicillin-resistant *Streptococcus pneumoniae* (PRSP) infection

* COVID-19 is designated as“pandemic influenza and relevant infections”, which is a different classification than categories I to V.

**Table 2:** Comparison of AMR bacteria prevalence based on hospital size, 2020 JANIS-CL annual report.

	Proportion of Patients with AMR Bacteria among Specimen-Submitting Patients (%)		
	All Hospitals	Hospitals with Number of Beds ≥200 Beds	Hospitals with Number of Beds <200 Beds
MRSA	6.41	5.93	10.1
MDRP	0.03	0.03	0.03
VRE	0.04	0.04	0.02
MDRA	0.00	0.00	0.00
CRE	0.31	0.34	0.13
Number of specimen-submitting patients			
	2,757,567	2,440,400	317,167
Number of participating hospitals			
	2167	1364	803

## Data Availability

The datasets described in this review are available on the website of the National Institute of Infectious Diseases (https://www.niid.go.jp/niid/ja/, accessed on 29 September 2021) and the JANIS official website in Japanese (https://janis.mhlw.go.jp/index.asp, accessed on 29 September 2021), and partly in English (https://janis.mhlw.go.jp/english/index.asp, accessed on 29 September 2021).

## Supplementary Materials

The following are available online at https://www.mdpi.com/article/10.3390/antibiotics10101189/s1, Table S1: Case definitions of AMR bacterial infections for NESID reporting.
