# Supplementary material for: A View on 20 Years of Antimicrobial Resistance in Japan by Two National Surveillance Systems: The National Epidemiological Surveillance of Infectious Diseases and Japan Nosocomial Infections Surveillance
Source: Antibiotics (Basel). 2021 Sep 30;10(10):1189. doi: 10.3390/antibiotics10101189 (PMC8532729; doi:10.3390/antibiotics10101189)
Supplement: Supplementary file 1 [file antibiotics-10-01189-s001.zip › antibiotics-1382838-supplementary.pdf]

Table S1 Case definitions of AMR bacterial infections for NESID reporting

For all AMR bacterial infection: (i) isolation of corresponding AMR bacteria from aseptic clinical specimens (e.g., blood, ascites, cerebrospinal fluid, etc.) or (ii) isolation of AMR bacteria from non-aseptic clinical specimens and clinical confirmatory diagnosis to ensure that the isolate is the causative infective pathogen. Each AMR bacteria must fulfil the requirements of laboratory results. AST, antimicrobial susceptibility test; MIC, minimum inhibitory concentration	
	Required laboratory results
MRSA	Isolation of bacteria identified as *Staphylococcus aureus* and the result of AST fulfil,<br>MIC of oxacillin ≥4 mg/mL or inhibitory zone diameter of oxacillin KB disk ≤10mm
MDRP	Isolation of bacteria identified as *Pseudomonas aeruginosa* and the result of AST fulfil all three,<br>(i) MIC of imipenem ≥ 16 mg/mL, or inhibitory zone diameter of imipenem KB disk ≤13mm<br>(ii)MIC of amikacin ≥32 mg/mL, or inhibitory zone diameter of imipenem KB disk ≤14mm<br>(iii) MIC of ciprofloxacin ≥4 mg/mL, or inhibitory zone diameter of ciprofloxacin KB disk ≤15mm
PRSP	Isolation of bacteria identified as *Streptococcus pneumoniae* and the result of AST fulfil, MIC of penicillin ≥ 0.125mg/mL or inhibitory zone diameter of oxacillin KB disk ≤19mm
VRSA	Isolation of bacteria identified as *Staphylococcus aureus* and the result of AST fulfil, MIC of vancomycin ≥ 16 mg/mL
VRE	Isolation of bacteria identified as *Enterococcus* sp. and the result of AST fulfil, MIC of vancomycin ≥ 16 mg/mL
MDRA	Isolation of bacteria identified as *Acinetobacter* sp. and the result of AST fulfil all three,<br>(i) MIC of imipenem ≥ 16 mg/mL, or inhibitory zone diameter of imipenem KB disk ≤13mm<br>(ii)MIC of amikacin ≥32 mg/mL, or inhibitory zone diameter of imipenem KB disk ≤14mm<br>(iii) MIC of ciprofloxacin ≥4 mg/mL, or inhibitory zone diameter of ciprofloxacin KB disk ≤15mm
CRE	Isolation of bacteria identified as Enterobacterales and the result of AST fulfil either<br>(i) MIC of meropenem ≥ 2 mg/mL, or inhibitory zone diameter of meropenem KB disk ≤22mm<br>(ii) MIC of imipenem ≥2 mg/mL, or inhibitory zone diameter of imipenem KB disk≤22mm<br>and MIC of cefmetazole ≥64 mg/mL, or inhibitory zone diameter of cefmetazole KB disk ≤22mm
